# The intersection of disability and food security: Perspectives of health and humanitarian aid workers

**DOI:** 10.4102/ajod.v7i0.322

**Published:** 2018-04-30

**Authors:** Candice A. Quarmby, Mershen Pillay

**Affiliations:** 1 Disciplines of Audiology & Speech-Language Therapy, University of KwaZulu-Natal, South Africa; 2 Discipline of Speech-Language Pathology, University of KwaZulu-Natal, South Africa

## Abstract

**Background:**

Most people with disabilities the world over can be found in the Majority (or ‘economically developing’) World. This is also where most of the world’s hungry and malnourished are found. We argue that the intersectionality between disability and nutrition may best be understood through a food security framework, and we position all people living with disability, including those experiencing feeding and swallowing disabilities, as at risk for food insecurity, especially those living in humanitarian emergency contexts.

**Objectives:**

This study aimed to explore and describe the knowledge and experience of humanitarian aid workers (HAWs) and health care professionals (HCPs) in food assistance contexts with regard to the nutrition and food security of people living with disabilities.

**Method:**

In this exploratory, descriptive study, 16 participants with experience in sub-Saharan Africa and Southern Asia participated in an online survey. Three survey participants with extensive experience were also interviewed. Data analysis involved descriptive statistics and thematic content analysis.

**Results:**

Results revealed that participants had generally low levels of exposure to and experience with disability, including swallowing and feeding disorders.

**Conclusions:**

Reduced knowledge of HAWs and HCPs regarding disability and the lack of professionals such as speech–language therapists, who manage disability-specific issues such as feeding and swallowing disorders, may affect the food security of people living with disabilities in food assistance contexts.

## Introduction

Eighty per cent of the one billion people living with disability the world over can be found in Majority (or low- and middle-income) World regions such as sub-Saharan Africa (World Health Organization & World Bank 2011). Similarly, the majority of the 794.6 million people worldwide who are estimated to be undernourished also live in these areas (Food and Agriculture Organization, World Food Programme & International Fund for Agricultural Development 2015). Therefore, it is a truism that people living with disabilities in vulnerable contexts may be predisposed to hunger and malnutrition. However, there is currently a poor understanding of the intersection of disability and malnutrition (Groce et al. 2013).

We believe that this connection may best be positioned as a food security concern, as in the case of people with disabilities, food security may be jeopardised by a range of issues from reduced mobility to the presence of feeding or swallowing disorders (dysphagia). Furthermore, we argue that the context in which disability-related issues may result in increased vulnerability to food insecurity is the same context in which vulnerability to food insecurity may be generally high and that individuals such as health care professionals (HCPs) and humanitarian aid workers (HAWs) who manage nutrition in these contexts (Filmer 2008) may have unique and valuable insight into this intersection.

## Disability and food security

Food is a fundamental human right (United Nations 1948). However, despite that there has been sufficient food to feed every person in the world for decades (Simon 2012), approximately one in every nine people worldwide has inadequate food to support a healthy, active life (FAO, WFP & IFAD 2014).

Food security is defined as the circumstance in which all people, at all times, have physical, social and economic access to sufficient, safe and nutritious food that meets their dietary needs and food preferences for an active and healthy life. (FAO et al. 2015:53)

The four dimensions of food security include food availability, access, utilisation and sustainability (FAO et al. 2015; Maxwell & Smith 1992; Webb & Rogers 2003). Currently, the dimension of access, which is the main focus of this article, is accepted globally to refer to physical, social and financial access to food (FAO et al. 2015). As can be seen from the fictional vignette below, which is rooted in the realities of Internally Displaced Persons (IDP) camps and conflict zones (International Organization for Migration 2014), these access classifications do not fully encompass the range of additional access issues that people living with disabilities encounter on a daily basis, especially in resource-poor regions such as sub-Saharan Africa.

Nwanneka is 6 years old and she and her family have lived in Yobe, a state in Northern Nigeria, for 1 year. They form part of a large group of IDP who fled their homes in Gujba because of violence at the hands of the Boko Haram insurgency. Nwanneka’s mother and five siblings collect food every day, but as Nwanneka cannot walk to the distribution site, nor carry the food home because of her severe spastic cerebral palsy, she relies on her family to collect her ration while she is left at home. Although it has never been diagnosed, Nwanneka presents with dysphagia, a swallowing disorder. This prevents her from efficiently and safely swallowing food and liquid – often leading to chest infections. In addition, she is unable to communicate verbally. Nwanneka cannot move her hand to her mouth, so her mother feeds her while she lies, contracted, on the floor of their hut in the IDP camp. Feeding Nwanneka is a difficult and time-consuming task for her mother. She coughs when she drinks water and her chest always sounds like a rattle. Nwanneka is generally fed only small amounts of food as, unlike her siblings who speak, she never complains of hunger. Anyway, she can only consume small amounts of food or liquid as most of it spills from her mouth during feeding.

Konje and Ladipo (2000), Kerac et al. (2014) and Wu et al. (2010) have reported malnutrition as a cause of disability. However, few researchers have explored alternative relationships between disability and nutrition such as the intersectionality between disability and feeding and swallowing. Feeding can be conceptualised as being reliant on physical, behavioural or cognitive access to food and liquid such as self-feeding skills. It also involves physiological access to food and liquid through swallowing. The ability to feed is therefore dependent on a number of different capabilities and functions, all of which may be impaired in different types of disabilities (Arvedson 2008; Cox et al. 2007). We argue that like other food access concerns such as reduced mobility or communication disorders resulting in reduced ability to request food, feeding and swallowing are significant when considering disability and nutrition. However, like other disability-specific access concerns, these issues have not yet been adequately linked. Thus, it is unsurprising that there was negligible reference in the World Report on Disability to people experiencing difficulties eating and drinking because of head injuries, strokes, developmental disabilities and suchlike (Pillay 2014; WHO & World Bank 2011).

Feeding difficulties can result from a large range of major motor and cognitive disabilities (Schwarz 2003), which can in turn lead to reduced food security. For example, 99% of individuals with cerebral palsy present with dysphagia (Calis et al. 2008), and more than 90% of individuals with motor neurone disease present with dysphagia (Hartelius & Svensson 1994). Specific feeding and swallowing disorders may include symptoms such as food refusal, overeating or aspiration (food or fluid entering the airways or lungs) as may be the case for people with stroke (Martino et al. 2005) or traumatic brain injury (Castano & Capdevila 2010; Terre & Mearin 2007). Furthermore, symptoms such as gastroesophageal reflux, dysphagia, prolonged feeding time and increased feeding-related caregiver stress have been identified in the cerebral palsy population (Adams et al. 2012; Schwarz 2003). People with visual impairment have been found to have difficulty shopping and preparing food (Muurinen et al. 2014) and may consume a diet lacking in variety as a result of inaccessibility of materials and environments (Bilyk et al. 2009). Elderly people with physical disabilities limiting mobility may not be able to access shops to buy food and have difficulties preparing food independently (Wylie, Copeman & Kirk 1999). These are just a few examples highlighting issues arising from both motor and sensory impairments which may limit the ability of a person living with a disability to access food and therefore to be food secure. People living with disabilities in impoverished or humanitarian emergency contexts may face even greater barriers to food access considering that disability-specific factors may be compounded further by environmental barriers in these vulnerable, resource-poor environments (Filmer 2008).

## Vulnerability to food insecurity

In the context of food insecurity, vulnerability is defined as the risk of future loss faced by an individual or a group as well as their incapacity to ensure adequate livelihood and food security over time (Woller et al. 2011). Vulnerability may be precipitated by a range of circumstances and is both widespread and complex (FAO et al. 2015). Vulnerability is also increased in resource-poor contexts, irrespective of region or the economic status of the general population (Coleman-Jensen, Nord & Singh 2013; Tarasuk, Mitchell & Dachner 2014). As risks to food security differ depending on the nature of vulnerability, so too does risk management depending on the specific risks. An example of a risk management strategy is food assistance. For approximately the last 50 years, food assistance has been a key strategy employed to deal with food insecurity in impoverished, vulnerable contexts (Simon 2012). Food assistance has a multitude of definitions, but for the purpose of this article is defined as the provision of food or cash for food-based purposes (Clay 2010). In this study, food assistance is used to highlight the circumstances of people living with disabilities and explore their possible risks to food security, even (and especially) within the context of risk management.

**FIGURE 1:**
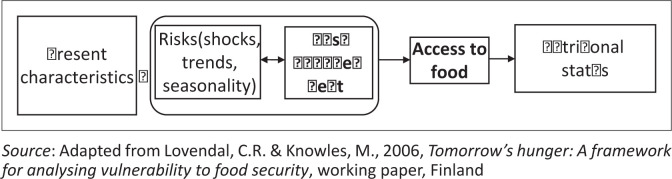
A framework for access to food in vulnerable contexts.

The framework in Figure 1 (Lovendal & Knowles 2006), which was used for this study, positions food security within a context of vulnerability, a common context for people living with disabilities. The framework highlights the effect of risks and risk management of vulnerable individuals or groups on the access dimensions of food security, which in turn affects nutritional status.

## Research methods and design

The design for this study was exploratory and descriptive in order to obtain insight, rather than focus on causal or contributory factors as in explanatory research (Creswell 2014). A mixed methods design was used, with a focus on qualitative data supplemented with quantitative data to increase the comprehensiveness of the study and to aid in the interpretation of data (Morse & Niehaus 2016). The study was conducted in four phases. Phase one included development of the research tools as well as pilot testing of the survey in order to improve the construction of the instrument (Creswell 2014). Phase two consisted of an online survey followed by three semi-structured interviews. Phases three and four involved data analysis and representation, respectively.

**FIGURE 2:**
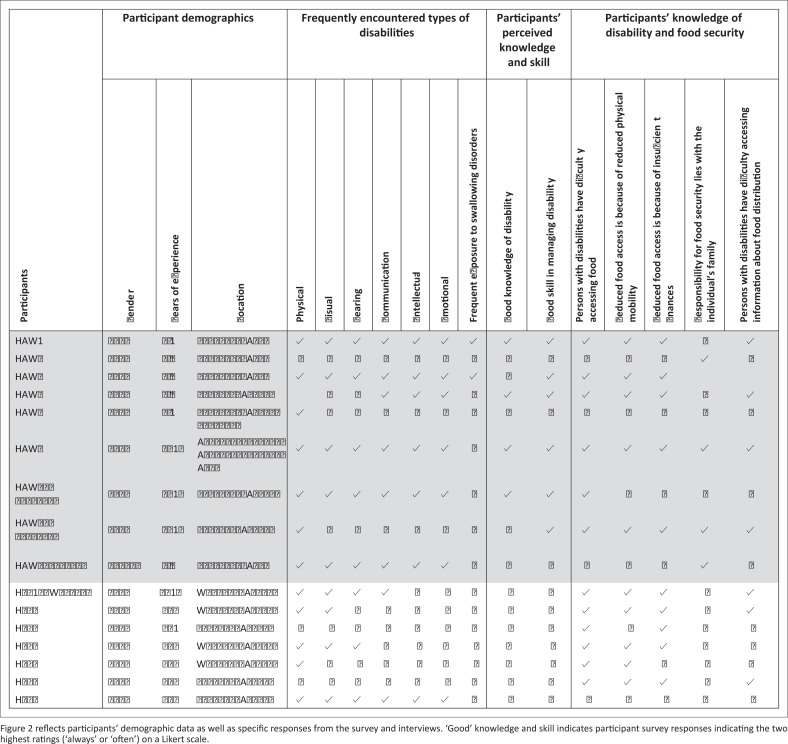
Summary of study results.

Recruiting participants via non-government organisation (NGO) gatekeepers was difficult. Once a gatekeeper had been established, despite frequent recruitment attempts in 14 different countries, limited participant responses were gained across the targeted study locations. Over the course of over 4 months, 16 HAWs and HCPs agreed to participate in the survey – all of whom had field experience in sub-Saharan Africa or Southern Asia and had worked with people living with disabilities (Figure 2).

The electronic questionnaire, an instrument developed specifically for this study and disseminated via Survey Monkey^®^, focused on (1) knowledge and experience of disability, (2) knowledge of the nutritional status of people living with disabilities and (3) knowledge of the food security status of people living with disabilities. It consisted of 17 closed-ended and 2 open-ended questions. Three survey participants, who were given the pseudonyms Jeremy, Edward and Mary, were then selected for in-depth interviews given their field experience (Figure 2). These three participants held managerial or leadership roles within their respective country organisations. Furthermore, they had more than 5–10 years of fieldwork experience in sub-Saharan Africa and Southern Asia. Thus, it was anticipated that these field experts would be able to provide insight into the circumstances of people living with disabilities who may be food insecure.

Survey results were extracted directly from Survey Monkey^®^ for analysis by a statistician using the Statistical Package for the Social Sciences (SPSS). Descriptive statistics were utilised (De Vaus 2014). Open-ended survey responses as well as interview data were analysed manually using thematic analysis (Miles et al. 2014). The researcher coded each data chunk, a colleague assisted with double-blinded coding of interview excerpts, and both sets of codes were compared for inter-coder agreement to improve dependability (Miles et al. 2014). Codes were reviewed and clustered into patterns, which depicted emergent themes in the data (Creswell 2014). The researcher then reviewed the themes, condensing the data into a cognitive, visual map depicting major themes and connecting inter-related themes. Additionally, certain codes and themes arising from the interviews were subjected to quantitative transformation, and the qualitative data were converted into quantitative data and subjected to frequency counts (Sandelowski 2000).

## Validity and reliability

As this research study was novel, the method and instruments were developed specifically for this study. The researchers attempted throughout the research process to assess and ensure data quality. Researchers ensured representativeness of findings by recruiting representative participants who, by nature of their work and locations, would provide relevant information regarding the concepts explored. To achieve construct validity, the research instruments were based on the specific aims and objectives of the study as well as the core concepts identified as relevant (Creswell 2014), which included dimensions of food security relating to disability and nutrition. These core concepts also guided data analysis. Construct validity was impacted initially by a greater focus on swallowing than on both swallowing and feeding. However, the interview schedule was revised during the research process (Miles et al. 2014) in order to include feeding as a concept and therefore strengthen validity. A pilot study was used to collect data regarding the clarity of the wording of the questions in the questionnaire in order to improve reliability of the final instrument (De Vaus 2014). Furthermore, inter-coder agreement (Creswell 2014) was observed when a second reviewer coded a portion of the qualitative data and minimal discrepancy was observed.

Confirmability and trustworthiness were ensured through triangulation of both data sources and research methods (Miles et al. 2014). Data sources included participants of different ages and gender with different roles. HCPs and HAWs were distributed over the wide range of countries in the two different study locations: sub-Saharan Africa and Southern Asia. Furthermore, the mixed methodology involved collection of data via survey with open- and closed-ended questions as well as interviews. Data analysis techniques such as statistical analysis as well as thematic analysis including triangulation strategies were also used. In this study, triangulation was used to develop in-depth, rich perspectives on disability, nutrition and food security from multiple angles.

## Results and discussion

The four major findings of the study described and discussed subsequently included knowledge of disability, knowledge of swallowing and feeding disorders, disability inclusion and caregiver involvement in the nutrition and food security of people living with disabilities.

### Knowledge and experience of disability

The study results (Tables 1 and 2) indicated that the participants had reduced knowledge and awareness of disability – both in general and for specific types of disabilities. This suggests that people living with disabilities may not be adequately managed. However, findings revealed that people with physical disabilities appear more likely to be managed than those with other types of disabilities. Physical disabilities were encountered often or always by 12 of the 16 survey respondents. Nine of the respondents indicated that they encountered visual disability often or always, while eight respondents reported that they encountered communication and hearing disabilities always or often in their fieldwork. Seven of the respondents indicated that they encountered intellectual and emotional disabilities often or always. Interview results revealed that all three interviewees, who had considerable field experience, had difficultly recalling specific experiences with people living with disabilities, and Mary indicated that only 11 of the 3500 registered children in her programme had a disability, further suggesting low exposure to disability. Considering that people with disabilities are at higher risk of food insecurity than people without disabilities (Huang, Guo & Kim 2009; Coleman-Jensen et al. 2013) and that the largest proportion of people with disability worldwide can be found in areas such as the study locations (WHO & World Bank 2011), these findings are concerning. Although the context of food aid provision has not been explored, similar results were found in an analysis of media coverage of food security and people with disabilities (Wolbring & Mackay 2014). This study found that the newspapers analysed largely excluded people with disabilities from the food security debate.

**TABLE 1:** Summary of participant responses.

Variables	Number of participants	Percentage of participants
**Type of disabilities frequently encountered by participants**		
Physical	12	75
Visual	9	56
Hearing	8	50
Communication	8	50
Intellectual	7	44
Emotional	7	44
Swallowing	2	13
**Participants’ perceived knowledge and skill**		
Good knowledge of disability	4	25
Good skill in managing disability	6	38
**Participants’ knowledge of disability and food security**		
Persons with disabilities have difficulty accessing food	13	81
Reduced food access is because of reduced physical mobility	10	63
Reduced food access is because of insufficient finances	10	63
Responsibility for food security lies with the individual’s family	4	25
Persons with disabilities have difficulty accessing information about food distribution	7	44

Table 1 reflects summary frequency and percentage counts of specific responses from the survey and interviews. ‘Good’ knowledge and skill indicates participant survey responses indicating the two highest ratings (‘always’ or ‘often’) on a Likert scale.

Physical disability was the most frequently encountered type of disability, a finding that may both promote and arise from the idea that a major aspect of food access is physical access. This may be the case as physical disabilities may be more visually obvious than with other types of disability. All three interviewees described experiences or scenarios with people with physical disabilities, and only Mary described another type of disability. Thirteen of the 16 respondents indicated that in their experience, people living with disabilities had reduced access to food. Ten of the respondents indicated that this was because of reduced physical mobility and reduced finances. These responses appear to reflect that people living with disabilities experience difficulties with access to food, as per FAO’s definition (FAO et al. 2015). However, other relevant issues people living with disabilities encounter remain unacknowledged. For example, people living with disabilities may have difficulty accessing information about planned food distribution because of a communication disorder or visual impairment (Muurinen et al. 2014). This may have an impact on the ability of a person living with a disability to access food. This was highlighted by Edward, another interviewee, both in the interview and in the survey when he reported ‘we sometimes forget that there are those who … cannot hear so as a result they miss important information (pertaining to food distribution)’ (HAW 8, male, from Eastern Africa). A further six survey respondents highlighted access to information as an issue for people living with disabilities. We believe that this failure to acknowledge a range of disability-related issues highlights a poor understanding of disability amongst HCPs and HAWs in food assistance contexts and in turn may place people living with disabilities at a greater risk of food insecurity. Furthermore, considering the varying and complex nature of disabilities, it seems unlikely that without exposure to different types of disabilities, HCPs and HAWs would be able to adequately manage their specific needs. It was therefore understandable when only four and six of the participants felt they often or always had adequate knowledge and skill (Table 1), respectively, to manage the nutrition, feeding and swallowing needs of people living with disabilities.

These results imply that people living with disabilities may be under-served in food assistance contexts. Fifteen per cent of the world’s adult population are estimated to present with a disability, and 5.7% of children are expected to have moderate to severe or severe disabilities (WHO & World Bank 2011). However, only 0.3% of the 3500 children in Mary’s example above were served; this indicates that a portion of children with disabilities was likely overlooked. This highlights a lack of knowledge of and exposure to disabilities amongst individuals who, by nature of their location and work, should encounter people living with disabilities more regularly than those in other contexts (WHO & World Bank 2011). A rigorous narrative literature review using systematic review principles, preferred reporting items for systematic reviews and meta-analyses (PRISMA) guidelines and double blinding (Liberati et al. 2009) was conducted for the purpose of this study. The findings supported the idea that people living with disabilities may not be adequately catered for or acknowledged in food assistance contexts (Duttine, Cherow & Farkas 2012; Quarmby 2016). An in-depth search of academic and grey literature published between 1985 and April 2015 aimed to identify if food assistance providers managed people living with disabilities living in vulnerable contexts. Of 1547 records identified, only 19 records discussed food assistance provision with overt reference to people living with disabilities, and only 2 records made explicit connections between disability and food security or nutrition in their conclusions (Duttine et al. 2012; Klesges et al. 2001), with 1 article indicating a scarcity of programmes addressing the nutritional needs of children with disabilities (Duttine et al. 2012). Both the scarcity of literature regarding disability and food security or nutrition and the findings of Duttine et al. (2012) corroborate the survey and interview findings that people living with disabilities may not be adequately catered for within food assistance contexts. This suspected oversight of people living with disabilities at organisational levels is perhaps unsurprising considering that in the World Report on Disability, reference to access to food for people living with disabilities is negligible, and only three indirect references to feeding and swallowing issues can be found (WHO & World Bank 2011).

### Knowledge and experience of swallowing and feeding disorders

Knowledge and experience of swallowing and feeding disorders amongst participants was low. Only two survey respondents reported that they often encountered people with swallowing disorders (Table 1). The remaining respondents never encountered swallowing disorders, or were unsure, and only one respondent highlighted this as a potential cause of reduced access to food for people living with disabilities. All three interviewees indicated that they had no experience with or knowledge of feeding or swallowing disorders, although Mary later recounted a story of a child with autism who had feeding difficulties. The prevalence of swallowing disorders has been estimated to be 16% of the general population in a high-income, urban context (Eslick & Talley 2008) and is therefore expected to be even higher in the Majority World. As feeding can be affected in a myriad of ways when a person presents with a disability (Adams et al. 2012; Bilyk et al. 2009; Castano & Capdevila 2010; Schwarz 2003), we consider reduced HCP and HAW knowledge and experience of feeding and swallowing disorders to be a risk factor that may contribute to reduced food access and therefore an increased risk of food insecurity for people living with disabilities. In terms of the conceptual framework (Figure 1), reduced HCP and HAW knowledge and experience of disability and feeding issues is likely to affect the quality and sufficiency of food assistance efforts. Irrespective of the availability of food or economic access issues (both of which may be accounted for in the context of food aid provision) or of the ability of an individual with disability to physically or socially access food assistance, which is also likely to be impaired (Poulsen et al. 2015), further sensory, cognitive and physical issues may impair access to food assistance through feeding. Feeding issues may, in turn, affect nutritional status. As such, the results indicating not only poor knowledge of feeding or swallowing disorders but also poor awareness of and exposure to all types of disabilities, especially sensory, cognitive and communication impairments, are especially concerning.

### Disability inclusion

It became evident during all three interviews that inclusion criteria for distribution programmes were governed by both internal and external policies. It also appeared that further independent decision-making by HAWs may have facilitated the inclusion of people living with disabilities as HAWs included those people observed to have disabilities at distribution points in the distribution efforts. Edward and Mary indicated that disability was included in the vulnerability criteria and people living with disabilities were therefore included in food distribution programmes. However, Jeremy highlighted informal identification and inclusion of people living with disabilities: ‘You notice there is someone that is living with a disability and you just call him’ (HAW 7, male, from Southern Africa). Edward indicated that people living with disabilities might be excluded from food distribution programmes and highlighted that this may be because of the insufficient nature of assessment of people living with disabilities. Although community involvement in the development of inclusion criteria and food aid programmes was highlighted by all three interviewees, Edward reported that programmes may not fully cater for people living with disabilities as the perspectives of people living with disabilities are not considered when planning such programmes: ‘when you are doing analysis of the information you will not be having the views of the disabled people’ (HAW 8, male, from Eastern Africa). Furthermore, neither Jeremy nor Mary discussed engaging people living with disabilities in programme development or execution.

Despite reported efforts to include people living with disabilities, again the general lack of knowledge and exposure evidenced by the study participants, when considered along with global disability statistics (WHO & World Bank 2011), suggests that people living with disabilities may not be sufficiently included in food aid programmes and may therefore be at risk of reduced access to food. Furthermore, disability inclusion may be impacted by disability management at a national level. At no point did any of the interviewees discuss state responsibility for the nutritional care or well-being of citizens with disabilities. In fact, this issue arose only once during the research process, when during the survey HCP1 indicated that a barrier to food security for people living with disabilities was that there was ‘no national program for food security for people with disability’ (Walter, male, from Western Africa). The Universal Declaration of Human Rights and the Convention for the Rights of Persons with Disabilities, both of which highlight the right to food, were signed and ratified by 185 and 160 member states, respectively, with good representation from sub-Saharan Africa and Southern Asia alike (UN 1948, 2015c). Similarly, almost 200 member states committed to halving extreme poverty and hunger through the millennium development goals (MDGs) (UN 2015a) and, more recently, to eliminating poverty and hunger by 2030 through the sustainable development goals (SDGs) (UN 2015b). These conventions and goals highlight global and national commitment to human rights, disability rights and food security. However, formal acknowledgement of rights by a state does not necessarily translate into policies or practices that promote these rights (FAO 2006) as can be seen in the lack of reports of government assistance for food security or people living with disabilities in the study locations. Despite MDG commitments, sub-Saharan Africa as a region showed significantly less progress with regard to poverty and hunger reduction than any other regions and was the only region that did not meet poverty and hunger goals (UN 2015c).

Reduced government involvement may lend itself to the self-regulation of humanitarian aid organisations (Lloyd 2005) that tend to adopt codes of ethics or accountability policies dictated by international organisations such as the World Association of Non-Government Organizations (WANGO). However, to our knowledge, these codes of ethical conduct or accountability do not account for people living with disabilities (The International Federation of Red Cross, Red Crescent Societies & the International Committee of the Red Cross 1994; WANGO 2007). This can be seen in the data through the scarcity of formalised organisational policies ensuring the inclusion and management of people living with disabilities, as well as through the reports of humanitarian aid worker autonomy to ensure disability inclusion.

### Caregiver responsibility

The results indicate a possible over-burdening of caregivers in terms of responsibility for the nutrition of people living with disabilities. Four participants indicated that the responsibility of assisting people living with disabilities to acquire food lay with the individual’s family (Figure 2), often a mother or grandmother, or community members, as was seen in survey responses from Edward and HAW2. Furthermore, Mary indicated that she believed that poor caregiver knowledge could cause ‘suffering’ for a child with a disability and suggested that it was the responsibility of the parent to provide food for their child with a disability,

‘so if the parents they know about that type of food they can normally provide them (children with disabilities) with that type of food and then the children become healthy.’ (HAW 9, female, from Southern Asia)

This quote highlights the expectations that caregivers are responsible for equipping themselves with knowledge and resources to adequately care for the person living with a disability. Once more, these data highlighted the issue of reduced food security through the mechanism of reduced food access, as not only does it appear that people living with disabilities are dependent on their caregivers for food, but these caregivers appear to bear the burden of disability and food insecurity where financial and time demands are great and resources are few.

The placement of responsibility on caregivers, specifically those in resource-poor contexts, hardly appears fair, especially considering that poverty-related issues such as diseases of poverty perpetuate poverty and vulnerability for these caregivers. Along with other disability-specific issues such as reduced income of persons living with disabilities as well as their carers (Huang et al. 2009), the capacity of caregivers in these contexts to adequately manage the nutrition and food security of their family member with a disability is reduced. This was highlighted by Mary who spoke of the lack of financial resources interfering with child care in that parents in her community needed to work and therefore did not have time, or energy, to adequately care for their children,

‘When her [*child with a disability*] mother back from her work- and that time she was tired … she cook for her family and she became tired. And just in the nights she tried to feeding her child.’ (HAW 9, female, from Southern Asia)

There appears, therefore, to be a mismatch between the expectations placed on caregivers and their capacity to manage the burden of disability, especially in resource-poor contexts.

In summary, the results suggested that HAWs and HCPs may have reduced exposure to all types of disabilities, especially those that are not visually obvious, which suggests reduced capacity of these individuals to adequately identify and manage people living with disabilities in the context of food assistance. Furthermore, it appears evident from the results that people living with disabilities may not be representatively included in food distribution efforts and that the responsibility for the nutrition and food security of people living with disabilities may be heavily skewed towards caregivers and family, those individuals who are likely in the least favourable position to take on such responsibility.

## Ethical considerations

The study was approved by the University of KwaZulu-Natal Humanities and Social Sciences Research Ethics Committee under the protocol reference number HSS/0306/015M.

## Limitations of the study

Representation of HCPs in the interviews may have contributed to a more rounded picture of the knowledge and experience of both participant groups. Furthermore, although feeding was addressed within the interviews, the results may have been richer with increased focus on feeding, rather than predominant focus on swallowing, in the online questionnaire, especially considering the significance of such impairments in the context of food security for people living with disabilities. Despite these limitations, the study methodology and design included strategies to uphold trustworthiness and confirmability, and the study therefore provides rich and valuable insights into food security for people living with disability.

## Recommendations

Organisational policy development across humanitarian aid organisations is recommended. Policy reform to include HAW and HCP training in disability and feeding is suggested. Through heightened awareness and knowledge of disability, it is anticipated that identification and therefore inclusion of people living with disabilities will improve, thereby promoting access to the food distributed. Furthermore, training in feeding may improve HAW’s or HCP’s ability to manage the nutrition of people living with disability and train or counsel caregivers appropriately. Although NGO resources may be scarce, it is recommended that these organisations prioritise the inclusion of HCPs such as speech–language therapists, occupational therapists or physiotherapists, all of whom are trained to practically manage people living with physical, sensory and cognitive disabilities.

Furthermore, inclusion of people living with disabilities in programme planning is recommended to promote the identification and management of specific barriers to food security that may be encountered by people living with disabilities.

Globally, there is an urgent need to reconsider food access within our current understanding of food security. This is important to account for risks to food access experienced by people living with disabilities and to uphold their fundamental right to food.

Further research exploring the specific circumstances of people living with disabilities in resource-poor contexts, particularly with regard to feeding and swallowing, is recommended to fully understand and confirm the mechanisms that put this vulnerable group at risk of poor access to food and food insecurity. Additionally, further exploration of these issues from the perspective of people with disability is essential in adding to the insights already gained from this study.

## Conclusion

These research findings suggest that people living with disabilities in resource-poor contexts, such as sub-Saharan Africa, may be at a greater risk of food insecurity because of reduced food access, specifically in the context of food assistance, where they may not be included or managed adequately.

More than anything, these results suggest that disability, nutrition and food security are interconnected and that these issues may be affected at a spectrum of levels ranging from the household and organisation levels to government and international levels. However, despite this intersection, the current understanding of food security fails to acknowledge disability-specific access issues, such as feeding or swallowing disorders, further impairing access to food and therefore food security for this vulnerable group of people.
