# Corrigendum: The intersection of disability and food security: Perspectives of health and humanitarian aid workers

**DOI:** 10.4102/ajod.v8i0.551

**Published:** 2019-10-10

**Authors:** Candice A. Quarmby, Mershen Pillay

**Affiliations:** 1 Discipline of Speech-Language Pathology, University of KwaZulu-Natal, South Africa

In the version of this article initially published, Candice A. Quarmby’s affiliation was incorrectly mentioned as Disciplines of Audiology & Speech-Language Therapy, University of KwaZulu-Natal, South Africa. Her correct affiliation is Discipline of Speech-Language Pathology, University of KwaZulu-Natal, South Africa. The error has been corrected in the PDF version of the article. The author apologises for any inconvenience that this omission may have caused.

