# Erratum: The intersection of disability and food security: Perspectives of health and humanitarian aid workers

**DOI:** 10.4102/ajod.v8i0.550

**Published:** 2019-10-10

**Authors:** Candice A. Quarmby, Mershen Pillay

**Affiliations:** 1 Discipline of Speech-Language Pathology, University of KwaZulu-Natal, South Africa

In the version of this article initially published, the data and text entries in Figures 1 and 2 were mistakenly published with omitted entries which presented them in an illegible format. The actual values for Figures 1 and 2 are updated and presented here:

**FIGURE 1:**
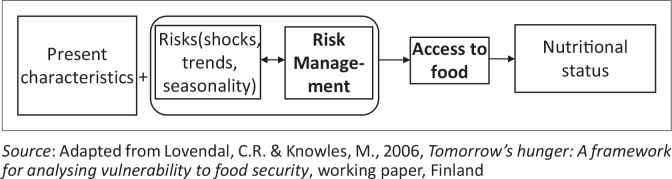
A framework for access to food in vulnerable contexts.

**FIGURE 2:**
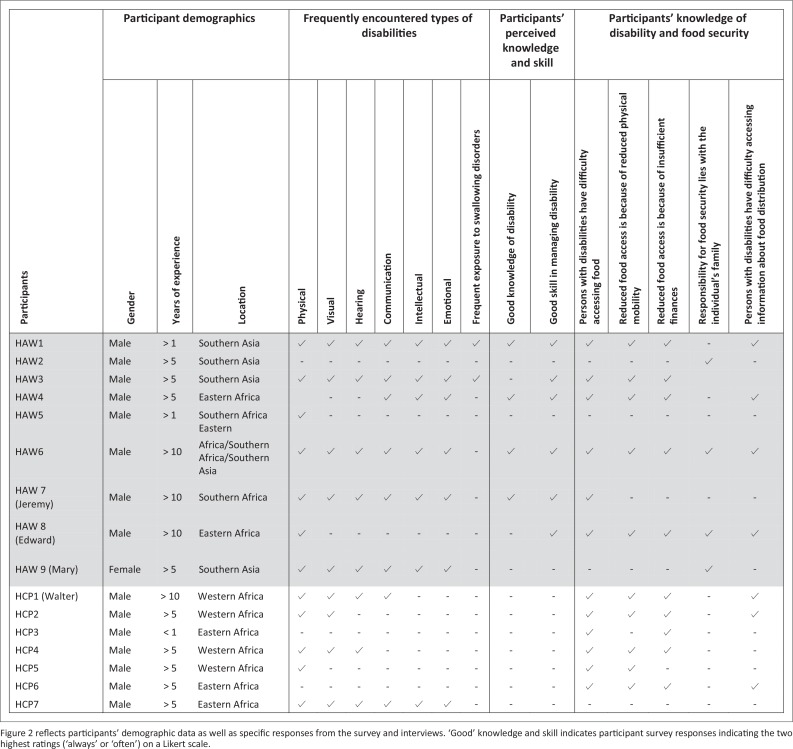
Summary of study results.

This correction does not alter the study’s findings of significance or overall interpretation of the study results. The errors have been corrected in the PDF version of the article. The publisher apologises for any inconvenience caused.

